# Editorial: Reviews in neuropsychology

**DOI:** 10.3389/fpsyg.2024.1367208

**Published:** 2024-04-23

**Authors:** María Jesús Luque-Rojas, Sara Palermo

**Affiliations:** ^1^Research Methods and Diagnosis in Education, Faculty of Education, University of Málaga, Málaga, Spain; ^2^Department of Psychology, University of Turin, Turin, Italy; ^3^Neuroradiology Unit, Department of Diagnostic and Technology, Fondazione IRCCS Istituto Neurologico Carlo Besta, Milan, Italy

**Keywords:** neuropsychology, autism spectrum disorder (ASD), false memory, decision making, integrative complexity theory, education

In the ever-evolving landscape of scientific inquiry, the discipline of neuropsychology stands as a beacon guiding our understanding of the intricate relationship between the human mind, behavior, and the underlying neural substrates. As we step into the new millennium, marked by unprecedented global challenges, the field of neuropsychology finds itself at the forefront of addressing the cognitive and behavioral impacts of the pandemic (Bartoli et al., 2020; Palermo, 2020) and post-pandemic era (Amanzio et al., 2021, 2022). The beginning of the 21st century has brought forth a myriad of challenges, notably exacerbated by the recent global pandemic. These challenges necessitate a reevaluation of our approaches to neuropsychological research and clinical applications. The disruptions caused by the pandemic have highlighted the urgency of understanding the profound impact of stress, isolation, and uncertainty on cognitive function and mental health (Morese and Palermo, 2022; Morellini et al., 2023).

*Reviews in Neuropsychology* is a Research Topic that serves as a testament of our commitment to advancing the frontiers of knowledge in this critical field. The collection of high-quality scholarly review articles within this Research Topics aims not only to showcase recent advances but also to illuminate unexplored avenues for future inquiries. The Research Topics demonstrate our dedication to exploring the association between cognitive-behavioral manifestations in both healthy and pathological subjects and their corresponding neural underpinnings. From clinical and observational tools to cutting-edge techniques such as electrophysiology and neuroimaging, our goal is to unravel the mysteries of the brain and its intricate interplay with cognition and behavior.

Beyond the traditional boundaries of neuropsychology, we extend our scope to encompass its intersection with education. This extension recognizes the importance of bridging the realms of neuroscience, neuropsychology, and education—a linkage ripe for exploration in the pursuit of a comprehensive understanding of human cognition.

The articles presented in this Research Topics will not only enrich the scholarly discourse within the neuropsychological community but also translate into tangible applications in clinical, public health, and policy settings. By fostering discussion and collaboration, the authors in this Research Topics aspire to propel the field of neuropsychology toward best practices that address the unique challenges of the new millennium.

This Research Topic comprises five contributions consisting of one editorial, three systematic reviews, and one meta-analysis.

In the Research Topic editorial, the authors trigger further debates within the neuropsychological community, with the hope that these discussions could translate into best practice applications in preclinical and clinical settings, as well as in public health and policy domains (Zhou et al., 2023).

The first review seeks to explore how individuals with mild cognitive impairment (MCI) perceive and process emotions, addressing a subject often overlooked in current literature. The results highlight a level of uncertainty, as some studies find no distinctions in emotion recognition and processing between the MCI and healthy control groups, while others identify specific deficits in recognizing both negative and neutral emotions in MCI patients (Morellini et al.).

The prevalence of comorbidity between epilepsy and autism spectrum disorder (ASD) in the pediatric population has significantly increased in recent years. This comorbidity negatively affects the cognitive-linguistic skills of individuals with ASD. The main objective of the second review is to examine the impact of epilepsy on the development of cognitive and linguistic skills in children with ASD (Cano-Villagrasa et al.). The study emphasizes that epilepsy in the ASD population leads to a reduction in cognitive and linguistic abilities. The impact varies based on different types of epilepsy and their locations, significantly affecting the quality of life and basic activities of daily living for individuals with ASD who also experience epilepsy.

The systematic review and meta-analysis examine the impact of substance abuse on false memory formation, an aspect less explored in the literature (Caetano et al.). Despite no noteworthy distinctions in false recognition/recall of critical lures observed between individuals with a history of substance abuse and those without such histories, those with a background of substance abuse exhibited notably elevated levels of false memories pertaining to both related and unrelated events.

A third review explores the relationship between decision-making outcomes and integrative complexity (IC) in various contexts (Molina et al.). Integrative complexity refers to the degree to which an individual considers multiple perspectives, dimensions, or aspects when making decisions or forming opinions. The analysis reveals research gaps in understanding the nature of IC, measurement challenges, and differentiation from other cognitive features. Opportunities for investigating brain activity during decision-making in relation to IC are identified. The discussion emphasizes the need for precise categorization of IC in cognition studies and explores implications for understanding the' cognitive nature of IC and the potential of neuroscience methods in studying this attribute.

Knowing the content and themes of each study included in this Research Topic makes us think about the need to include and consider the area of neuropsychology in many diverse contexts, highlighting one of them: Education. Educational neuroscience and neuropsychology are an interdisciplinary field of research (neuroscience, education, and psychology) (Dennis, 2019) that seeks to understand the effects of education on the brain (Han et al., 2019; Thomas et al., 2019; Vaughn et al., 2020). For some time now, education has needed insights from neuropsychology so that all professionals who are part of education have the tools to teach from the learner's brain. Consequently, including many of the results and contributions of the studies in Neuropsychology in Education, research in the field of neuroscience and neuropsychology favors, to a large extent, our understanding of the teaching-learning processes, providing a much more solid basis on which to base the steps to be taken in education (Wolfe, 2010).

Pickering and Howard-Jones (2007) concluded that teachers showed great interest in the combination of neuroscience, neuropsychology, and education, considering it necessary to develop programs where the brain and its functioning are the basis of development understanding, in addition to believing in the importance of transferring neuroscience content to teachers to help them better understand certain practices in the classroom.

In conclusion, this compilation of research not only expands our understanding of neuropsychology but also highlight the field's dynamic response to contemporary challenges. The diverse perspectives offered in these reviews pave the way for future research, collaboration, and the application of findings in real-world settings, thereby shaping the trajectory of neuropsychology in the years to come.

## Author contributions

ML-R: Writing – review & editing, Writing – original draft, Conceptualization. SP: Writing – original draft, Writing – review & editing, Conceptualization.
